# Dietary NiCl_2_ causes G_2_/M cell cycle arrest in the broiler's kidney

**DOI:** 10.18632/oncotarget.5934

**Published:** 2015-09-30

**Authors:** Hongrui Guo, Hengmin Cui, Xi Peng, Jing Fang, Zhicai Zuo, Junliang Deng, Xun Wang, Bangyuan Wu, Kejie Chen, Jie Deng

**Affiliations:** ^1^ Key Laboratory of Animal Diseases and Environmental Hazards of Sichuan Province, Ya'an, China; ^2^ College of Veterinary Medicine, Sichuan Agricultural University, Ya'an, China

**Keywords:** NiCl_2_, G_2_/M cell cycle arrest, mRNA expression, protein expression, kidney

## Abstract

Here we showed that dietary NiCl_2_ in excess of 300 mg/kg caused the G_2_/M cell cycle arrest and the reduction of cell proportion at S phase. The G_2_/M cell cycle arrest was accompanied by up-regulation of phosphorylated ataxia telangiectasia mutated (p-ATM), p53, p-Chk1, p-Chk2, p21 protein expression and ATM, p53, p21, Chk1, Chk2 mRNA expression, and down-regulation of p-cdc25C, cdc2, cyclinB and proliferating cell nuclear antigen (PCNA) protein expression and the cdc25, cdc2, cyclinB, PCNA mRNA expression.

## INTRODUCTION

Nickel (Ni) is a metal widely distributed in the environment and is necessary for many industrial and commercial uses [1-3]. As important materials in many processes of modern industries, such as electroplating, welding and alloy production, Ni and Ni compounds may be released into the environment with relatively high amounts at all stages of production, recycling and disposal [4]. Also, Ni is a nutritionally essential trace metal for several animal species, micro-organisms and plants, and is a constituent of enzymes and proteins at low amount, but Ni at higher concentrations is toxic and carcinogenic to many organisms [1, 5, 6].

Epidemiological studies of Ni compounds from occupationally exposed populations have accumulated considerable evidences that exposure to both water-insoluble and water-soluble Ni is associated with lung and nasal cancers [7, 8], and Ni is therefore considered as a carcinogen [4, 9]. Our own studies [10-13] have also shown that dietary NiCl_2_ in excess of 300 mg/kg can cause immunotoxicity, oxidative damage and apoptosis in the kidney, spleen, small intestines and cecal tonsil of broiler chickens. Interestingly, Ni nanowires (Ni NWs) have been found to induce cell cycle arrest and apoptosis by generation of reactive oxygen species (ROS) in HeLa cells [14]. A novel Ni(II) thiosemicarbazone complexes encourage ROS hyper-generation with subsequent depletion of intracellular antioxidant pool, and mitochondrial membrane depolarization leads to caspase activation and DNA fragmentation which are the hallmarks of apoptosis [15].

It is well known that cell cycle includes S (DNA replication), M (nuclear division and cell division), G_1_ (the cell-cycle gap phase between M phase and S phase), G_2_ (the cell-cycle gap phase between S phase and M phase) phases, which is central to maintain homeostasis in the multicellular organisms [16]. Loss of cell cycle control may lead to imbalances in cell proliferation and cell death that contribute to various disease states including tumor formation [17, 18]. In response to various types of DNA damages, the cell cycle regulatory molecules and cell death signals are activated to stop cell growth and to eliminate multiplication of genetically altered cells [19]. The G_1_ and G_2_ phases in the cell cycle play very important roles as checkpoints in the regulation of cells proceeding to S and M phases, respectively [19]. In damaged cells, the cycle pauses longer in the G_1_ and G_2_ phases, which provides more time for repair of DNA damage before completing the next round of cell division. Prolonged cell-cycle arrest can induce growth arrest or apoptosis [20-22]. Shiao et al. [23] report that Ni acetate can increase the cell percentages in G_2_/M phase and apoptosis in Chinese hamster ovary cells. Ma et al. [14] suggest that Ni NWs significantly increase cell percentages in S phase in HeLa cells, and our previous study suggests that NiCl_2_ induces cell-cycle arrest at the G_0_/G_1_ phases in thymus [24].

Although there are some studies on cell-cycle arrest induced by Ni- and Ni compounds, few reports focus on the relationship between NiCl_2_ and cell cycle in the kidney. Therefore, the objective of this study was to investigate how NiCl_2_ induced cell cycle arrest. The commercial broilers' growth cycle is about 42 days, and then they will be put into use for consumption. In this period they grow rapidly and a lot of diet will be consumed, and broilers will easily affected by diet containing metal pollutants (such as Ni). The aim of our study is to evaluate the effect of dietary NiCl_2_ on the broilers in the period of growth. At 14, 28 and 42 days of the experiment, we monitored the cell cycle arrest in the kidney of broiler chickens fed on diets supplemented with various amounts of NiCl_2_. The protein expression of phosphorylated ataxia telangiectasia mutated (p-ATM), p53, p-Chk1, p-Chk2, p21 p-cdc25C, cdc2, cyclin B and proliferating cell nuclear antigen (PCNA) protein expression and the mRNA expression levels of nine genes involved in G_2_/M transition: ATM, p53, p21, Chk1, Chk2, cdc25, cdc2, cyclin B, PCNA were detected.

## RESULTS

### The cell cycle changes in the kidney

**Figure 1:**
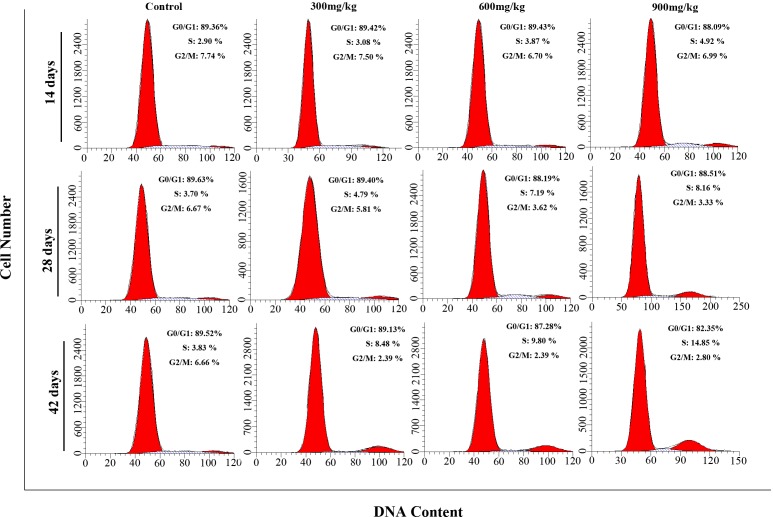
Cell cycle changes in the kidney by flow cytometry

**Figure 2:**
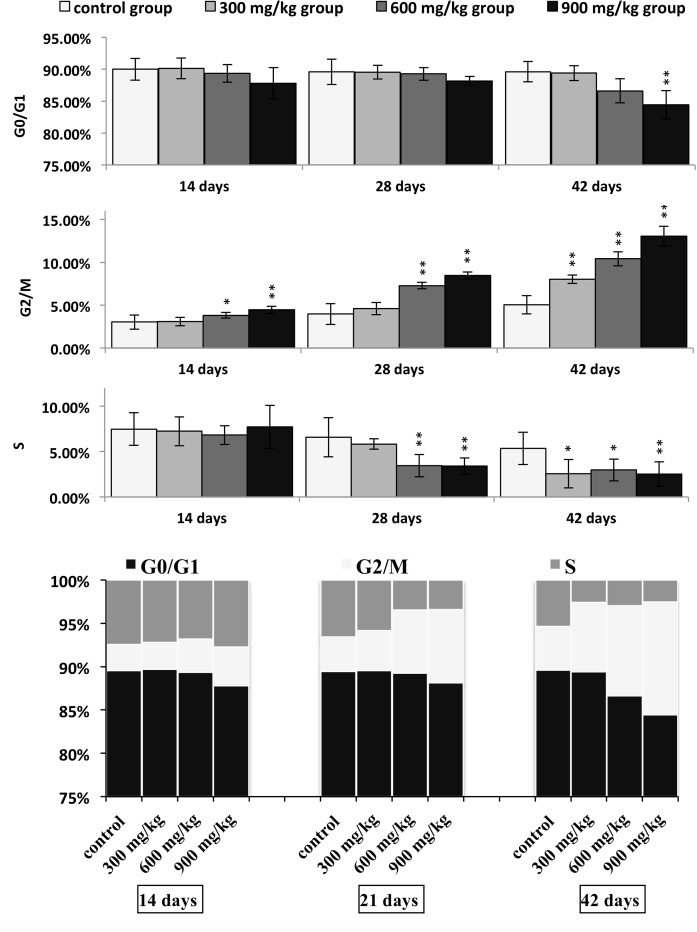
Changes of cell cycle phase distribution (%) in the kidney Data are presented with the mean ± standard deviation (n=5) *P<0.05, compared with the control group **P<0.01, compared with the control group.

Figures 1 and 2 show a dose and time dependent increase in G_2_/M phase cells and a corresponding decrease in cells at other stages of the cell cycle.

The cell percentages in G_0_/G_1_ phase were significantly decreased (*P* < 0.05 or *P* < 0.01) in the 900 mg/kg groups at 42 days of age when compared with those in the control group. The cell percentages in G_2_/M phase were significantly increased (*P* < 0.05 or *P* < 0.01) in the 600 and 900 mg/kg groups from 14 to 42 days of age and in the 300 mg/kg groups at 42 days of age in comparison with those in the control group. The cell percentages in S phase were significantly lower (*P* < 0.05 or *P* < 0.01) in the 600 and 900 mg/kg groups at 28 days of age and in the three NiCl_2_-treated groups at 42 days of age than those in the control group.

### Changes of the cell cycle regulatory molecule protein expression in the kidney

**Figure 3:**
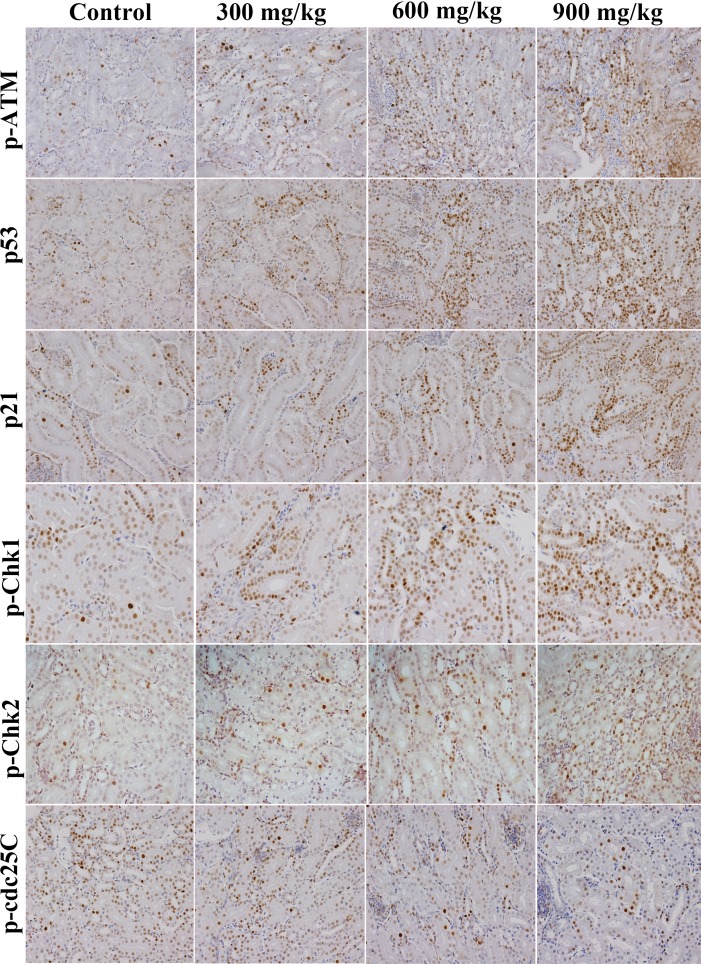
Changes of p-ATM, p53, p21, p-Chk1, p-Chk2 and p-cdc25C protein expression levels in the kidney at 42 days of age (Immunohistochemistry, ×400).

**Figure 4:**
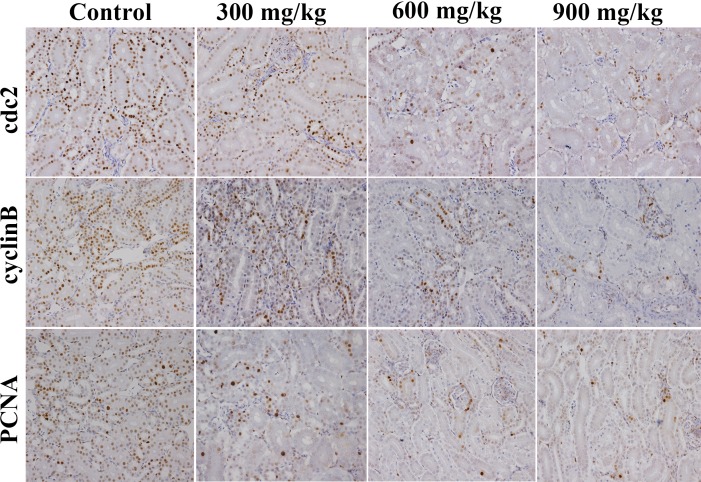
Changes of cdc2, cyclinB and PCNA protein expression levels in the kidney at 42 days of age (Immunohistochemistry, ×400).

**Figure 5:**
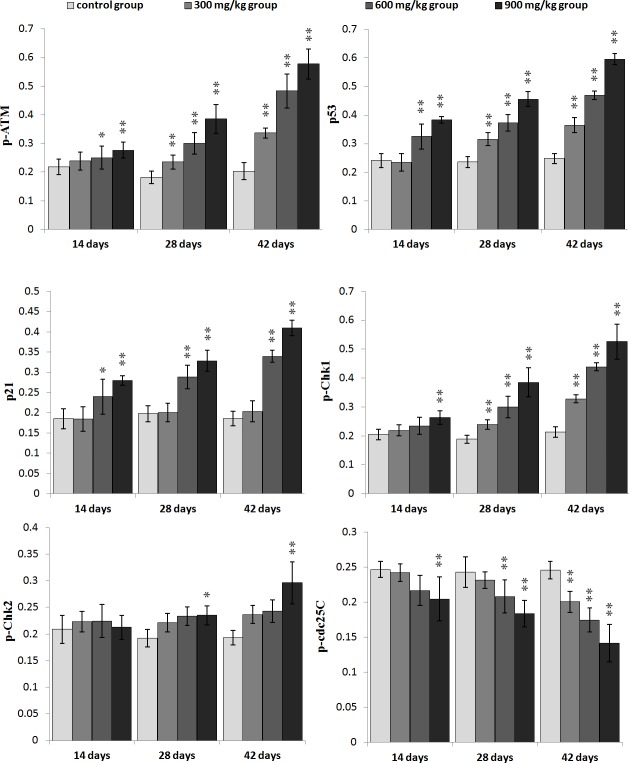
Changes of the mean density of p-ATM, p53, p21, p-Chk1, p-Chk2 and p-cdc25C protein expression in the kidney Data are presented with the mean ± standard deviation (n=5×5) *P<0.05, compared with the control group **P<0.01, compared with the control group.

**Figure 6:**
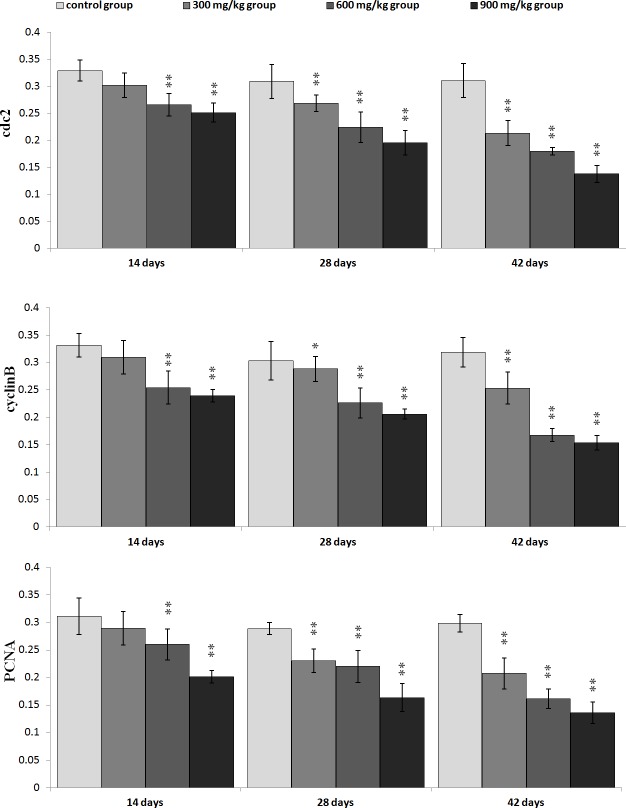
Changes of the mean density of cdc2, cyclinB and PCNA protein expression in the kidney Data are presented with the mean ± standard deviation (n=5×5) *P<0.05, compared with the control group **P<0.01, compared with the control group.

The changes of G_2_/M cell cycle regulatory molecule protein expression are shown in Figures 3, 4, 5, 6.

The p-ATM and p53 protein expression was significantly increased (*P* < 0.05 or *P* < 0.01) in the 600 mg/kg and 900 mg/kg groups at 14 days of age and in the three NiCl_2_-treated groups from 28 to 42 days of age when compared with those in the control group. The p21 protein expression was significantly higher (*P* < 0.05 or *P* < 0.01) in the 600 mg/kg and 900 mg/kg groups from 14 to 42 days of age than those in the control group. The p-Chk1 protein expression was significantly increased (*P* < 0.05 or *P* < 0.01) in the 900 mg/kg groups at 14 days of age and in the three NiCl_2_-treated groups from 28 to 42 days of age in comparison with those in the control group. The p-Chk2 protein expression was significantly higher (*P* < 0.05 or *P* < 0.01) in the 900 mg/kg groups than that in the control group from 28 to 42 days of age. The p-cdc25C protein expression was significantly decreased (*P* < 0.01) in the 900 mg/kg groups at 14 days of age, and in the 600 and 900 mg/kg groups at 28 days of age, and in the 300, 600 and 900 mg/kg groups at 42 days of age.

The cdc2, cyclin B and PCNA protein expression was significantly lower (*P* < 0.05 or *P* < 0.01) in the 600 mg/kg and 900 mg/kg groups at 14 days of age and in the three NiCl_2_-treated groups from 28 to 42 days of age than those in the control group.

### Changes of cell cycle regulatory molecule mRNA expression in the kidney

**Figure 7:**
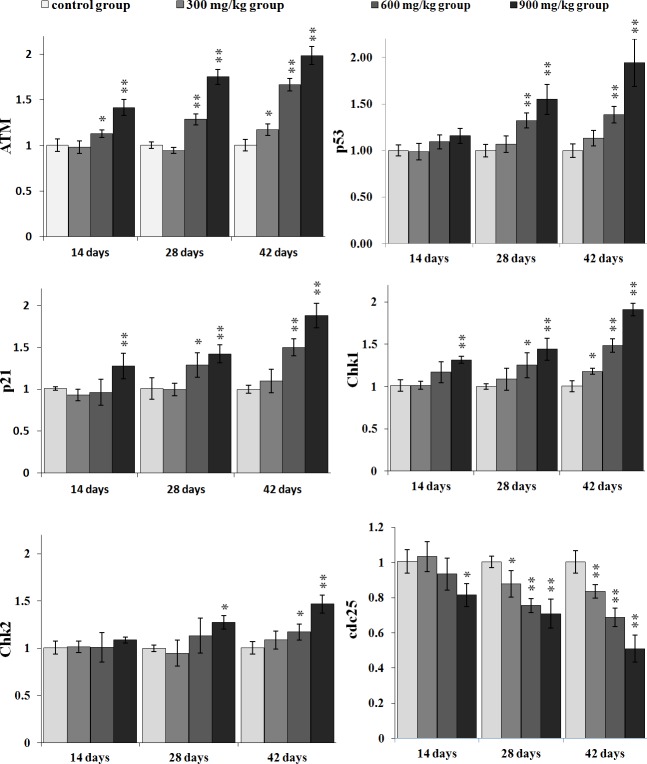
Changes of ATM, p53, p21, Chk1, Chk2 and cdc25 mRNA expression levels in the kidney Data are presented with the mean ± standard deviation (n=5) *P<0.05, compared with the control group **P<0.01, compared with the control group.

**Figure 8:**
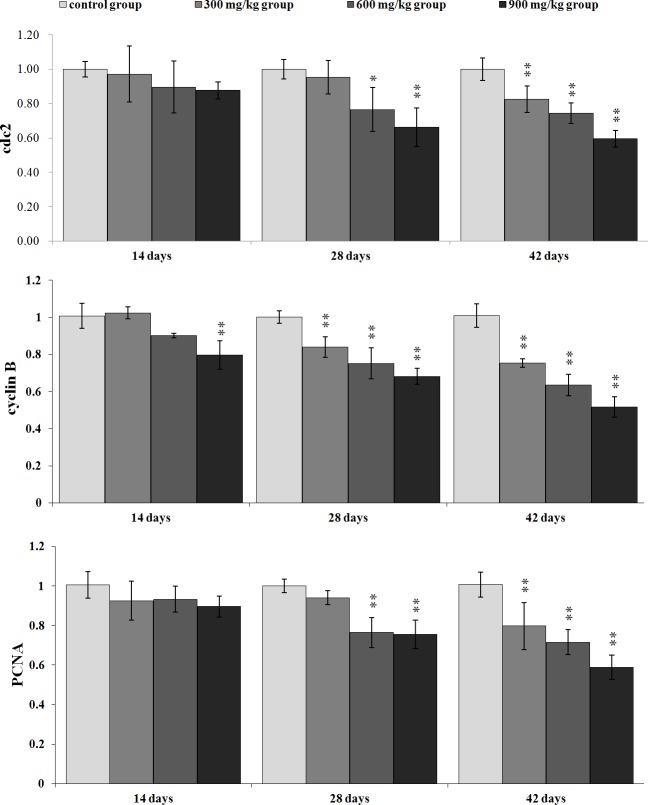
Changes of cdc2, cyclinB and PCNA mRNA expression levels in the kidney Data are presented with the mean ± standard deviation (n=5) *P<0.05, compared with the control group **P<0.01, compared with the control group.

Figures 7 and 8 show that changes of the G_2_/M cell cycle regulatory molecule mRNA expression levels are consistent with the changes of protein expression levels.

The ATM mRNA expression was significantly increased (*P* < 0.05 or *P* < 0.01) in the 600 mg/kg and 900 mg/kg groups from 14 to 42 days of age and in the 300 mg/kg group at 42 days of age when compared with that in the control group.

The mRNA expression of p53 and p21 was higher (*P* < 0.05 or *P* < 0.01) in the 600 mg/kg and 900 mg/kg groups from 28 to 42 days of age than that in control group, and the mRNA expression of p21 were increased (*P* < 0.05 or *P* < 0.01) in the 900 mg/kg group at 14 days of age.

The Chk1 mRNA expression was significantly increased (*P* < 0.05 or *P* < 0.01) in the 900 mg/kg groups at 14 days of age, and in the 600 and 900 mg/kg groups at 28 days of age, and in the 300, 600 and 900 mg/kg groups at 42 days of age. The Chk2 mRNA expression was significantly higher (*P* < 0.05 or *P* < 0.01) in the 900 mg/kg group at 28 days of age and in the 600 and 900 mg/kg groups at 42 days of age than that in the control group. The mRNA expression of cdc25 was significantly lower (*P* < 0.05 or *P* < 0.01) in the three NiCl_2_-treated groups at 28 and 42 days of age than that in the control group and was significantly decreased (*P* < 0.05 or *P* < 0.01) in the 900 mg/kg group at 14 days of age.

The mRNA expression of cdc2 and cyclin B was significantly lower (*P* < 0.05 or *P* < 0.01) in the three NiCl_2_-treated groups at 28 and 42 days of age than that in the control group, except for Cdc2 in the 300 mg/kg group at 28 days of age. Also, the mRNA expression of cyclin B was significantly decreased (*P* < 0.05 or *P* < 0.01) in the 900 mg/kg group at 14 days of age.

The PCNA mRNA expression was significantly decreased (*P* < 0.05 or *P* < 0.01) in the three NiCl_2_-treated groups at 28 and 42 days of age when compared with that in the control group, except for in the 300 mg/kg group at 28 days of age.

## DISCUSSION

This study shows the molecular control pathways of dietary NiCl_2_-induced the cell cycle arrest in the kidney of broiler chickens. We find consistent evidence that dietary NiCl_2_ in excess of 300 mg/kg has adverse effects on the renal cells. It induces cell cycle arrest at the G_2_/M phase, which results in cell-growth arrest or apoptosis when damaged cells can't be repaired [25]. Diets containing NiCl_2_ also decreased cell percentages in S phase. (Figures 1 and 2)

Our results are consistent with the report of Shiao et al. [23] that nickel acetate increases the cell proportion in G_2_/M phase and decreases the cell proportion in S phase in the Chinese hamster ovary cells. The reduction of cell percentages in S phase found in this study is also in agreement with the results that Ni(II)-containing media reduce cell percentages in S phase in murine L-929 fibroblasts, human gingival fibroblasts and human tissue mast cells [26]. However, there also is a report that Ni NWs increase cell percentages in S phase in HeLa cells [14].

In order to define how dietary NiCl_2_ induced G_2_/M cell cycle arrest in the kidney, we measured G_2_/M phase cell cycle regulatory molecules. The results showed that NiCl_2_ increased the p-ATM, p-Chk1, p-Chk2 and p53 protein expression, and ATM, Chk1, Chk2 and p53 mRNA expression (Figures 5 and 7). Salnikow et al. [27] have reported that NiCl_2_ and nickel sulfide (Ni_3_S_2_) can elevate the levels of p53 protein in human lung cell. There is a similar result that NiCl_2_ increases p53 protein levels in MCF-7 and A549 cells [28]. The central position of ATM in the maintenance of genomic stability becomes apparent by its involvement in checkpoint regulation at the G_2_/M transition [29, 30]. From the results in the present study, it is certain that NiCl_2_ activates the ATM signal transduction pathways by up-regulation of ATM and of its multiple downstream targets including Chk1, Chk2 and p53 [31, 32]. Further, NiCl_2_ induces G_2_/M phase arrest *via* two different routes: the ATM-p53 and ATM-Chk1/Chk2 pathways. These findings are similar to the report that cadmium chloride (CdCl_2_) has been shown to cause G_2_/M phase arrest in NRK-52E cells *via* elevation of p53 activity, up-regulation of cyclin kinase inhibitors p27 and p21 expression, and down-regulation of cyclin-dependent kinases Cdk1, Cdk2, cyclin A and cyclin B expression [33].

p53-dependent G_2_/M cell cycle arrest has also been observed upon exposure to arsenic trioxide (ATO) and CdCl_2_ in myeloma cells and rat renal proximal epithelial cells [34]. Thus, p53 appears to be a critical mediator of at least two or more cellular responses to a variety of DNA damage: apoptosis, DNA repair and cell cycle arrest [35], and p53-dependent G_2_/M cell cycle arrest is an important component of the cellular response to stress [31, 36]. It is also known that p53 can induce p21 up-regulation, which causes cells to arrest in G_2_/M phase [37]. Our results show that NiCl_2_ increases p21 protein and mRNA expression, and decreases cyclin B, cdc2, PCNA protein and mRNA expression. These results are consistent with several studies that different stimuli can induce cell cycle arrest through p53-dependent p21 activation [38-40]. Overall, p21 is known to induce cell cycle arrest at G_2_ by inhibiting cdc2 [41-43]. An inactive cdc2/cyclin B complex does not allow cells to progress beyond the G_2_/M cell-cycle checkpoint. At the same time, p21 inhibits DNA replication and maintains G_2_/M cell cycle arrest through the reduction of PCNA [37, 44-46]. The PCNA gene product acts as an auxiliary factor for DNA polymerase and stimulates DNA replication [47]. The limitation of cdc2/cyclin B complex formation and the down-regulation of PCNA expression block the passage of cells to mitosis [48, 49]. The results obtained here are consistent with the study that β-Mangostin can cause the p53-dependent G_2_/M cell cycle arrest by down-regulating cdc2 and PCNA [50].

Concurrently, up-regulation of p-Chk1, p-Chk2 protein and Chk1, Chk2 mRNA expression indicates that NiCl_2_ also activates ATM-Chk1/Chk2 pathways to inhibit cdc2/cyclin B expression. The reduction of cdc2/cyclin B complex induces G_2_/M cell cycle arrest. Joe et al. [51] and Yoda et al. [52] have reported that ATO can increase the protein expression of p-Chk1 and p-Chk2 in mice and NB4, HL-6 cells. It has also been noted that Chk1 and Chk2 are required for the initiation and maintenance of DNA damage-induced G_2_/M cell cycle arrest [53, 54]. Further, Chk1 and Chk2 inhibit cdc2 *via* inactivating cdc25, which can otherwise activate cdc2 trough the phosphatase [55-57]. Our results show that NiCl_2_ reduces the protein expression of p-cdc25C and the mRNA expression of cdc25. And some studies have also shown that Chk2 can induce G_2_/M cell cycle arrest through p53 up-regulation [54, 58].

In conclusion, dietary NiCl_2_ in excess of 300 mg/kg causes the G_2_/M cell cycle arrest in the broilers kidney, which is accompanied by the increase of ATM, p53, p21, Chk1, Chk2 protein and mRNA expression, and decrease of cdc25, cdc2, cyclin B, PCNA protein and mRNA expression.

## MATERIALS AND METHODS

### Experimental design

Two hundred and eighty one-day-old healthy broilers were divided into four groups. There were seventy broilers in each group. Broilers were housed in cages with electrical heaters, and provided with water as well as under-mentioned experimental diets *ad libitum* for 42 days.

To observe the time-dependent dynamic change, we chose three time points (14, 28 and 42 days of age) for examining cell cycle, and G2/M cell cycle regulatory molecule protein expression and mRNA expression levels.

In this study, a corn-soybean basal diet formulated by the National Research Council [59] was the control diet. NiCl_2_ (NiCl_2_·6H_2_O, ChengDu Kelong Chemical Co., Ltd., Chengdu, China) was mixed into the corn-soybean basal diet to produce the experimental diets containing 300, 600 and 900 mg/kg NiCl_2_, respectively.

The basis of doses (300, 600 and 900 mg/kg NiCl_2_) selection: Ling and Leach reported that dietary NiCl_2_ concentrations of 300 mg/kg and over resulted in significant reduction in growth rate. Mortality and anemia were observed in chicks receiving 1100 mg/kg nickel [6]. Weber and Reid found a significant growth reduction at 700 mg/kg NiSO_4_ and nickel acetate and over [60]. Chicks fed on more than 250-300 mg/kg Ni in the diet exhibited depressed growth and reduced feed intake [61]. Bersenyi et al. [62] reported that supplementation of 500 mg/kg NiCl_2_ reduced weight gain (by 10%), feed intake (by 4%) and worse FCE (by 5%) in growing broiler cockerels. According to the above-mentioned research results and our preliminary experiment, we chose the doses of 300, 600 and 900mg/kg NiCl_2_ in this study for observing the does-dependent changes.

Our experiments involving the use of broilers and all experimental procedures were approved by Animal Care and Use Committee, Sichuan Agricultural University.

### Cell cycle analysis by flow cytometry

At 14, 28, and 42 days of age, five broilers in each group were taken for determination of the cell-cycle stages in the kidney by flow cytometry.

The method of Cui et al. [63] was used and performed as described by the original authors. Briefly, the chickens in each subsample were humanely killed, and their kidneys were immediately taken and ground to form a cell suspension, which was filtered through a 300-mesh nylon screen. The cells were washed twice with ice-cold phosphate buffer saline (PBS, pH 7.2-7.4), and then suspended in PBS at a concentration of 1 × 10^6^ cells/mL. A total of 500 μL of the cell suspension was transferred to a 5-mL culture tube. After centrifugation (600 rpm, 5 min), the supernatant was decanted, the cells were incubated for 30 min at room temperature in the dark with 5 μL 0.25% Triton X-100 and 5 μL Propidium Iodide (PI) (Cat. No.51-66211E). Finally, 500 μL of PBS were added to each tube, and cells were analyzed by flow cytometry (BD FACSCalibur) within 45 min of preparation. The results were analyzed using the Mod Fit LT for Mac V3.0 computer program.

### Determination of the cell cycle regulatory molecule protein expression by immunohistochemistry

Five chickens in each group were humanely sacrificed for gross examination at 14, 28 and 42 days of age. Kidneys were collected and fixed in 4% paraformaldehyde, dehydrated in ethanol and embedded in paraffin.

The method was described by Wu et al. [11]. Kidney slices were dewaxed in xylene, rehydrated through a graded series of ethanol solutions, washed in distilled water and PBS and endogenous peroxidase activity was blocked by incubation with 3% H_2_O_2_ in methanol for 15 min. The sections were subjected to antigen retrieval procedure by microwaving in 0.01 M sodium citrate buffer pH 6.0. Additional washing in PBS was performed before 30 min of incubation at 37°C in 10% normal goat serum (Boster, Wuhang, China). The slices were incubated overnight at 4°C with the primary antibodies (Table 1). After washing in PBS, the slices were exposed to 1% biotinylated goat anti-rabbit/mouse IgG secondary antibody (Boster, Wuhang, China) for 1 h at 37°C, and then incubated with strept avidin-biotin complex (SABC; Boster, Wuhang, China) for 30 min at 37°C. To visualize the immunoreaction, sections were immersed in diaminobenzidine hydrochloride (DAB; Boster, Wuhang, China). The slices were monitored microscopically and stopped by immersion in distilled water, as soon as brown staining was visible. Slices were lightly counterstained with hematoxylin, dehydrated in ethanol, cleared in xylene and mounted.

**Table 1:** Antibodies used in immunohitochemistry

Name	Company	Cat#	Dilution
p-ATM	Bioss, China	bs-2272R	1:100
p-Chk1	Bioss, China	bs-5251R	1:100
p-Chk2	Bioss, China	bs-3721R	1:100
p53	Boster, China	BM0101	1:100
p21	Boster, China	BA0272	1:100
p-cdc25C	Bioss, China	bs-3482R	1:100
p-cdc2	Boster, China	BM0027	1:100
cyclinB1	Bioss, China	bs-0572R	1:100
PCNA	Boster, China	BM0104	1:100

The cell cycle checkpoint protein expression was counted using a computer-supported imaging system connected to a light microscope (OlympusAX70) with an objective magnification of ×400. The mean intensity of staining for each protein was quantified using Image-pro Plus 5.1 (USA) as described previously. Each group was measured five sections and each section was measured five visions and averaged.

### Determination of the cell cycle regulatory molecule mRNA expression by quantitative real-time PCR

The kidneys from five chickens in each group were taken at 14, 28, and 42 days of age and stored in liquid nitrogen. They were then homogenized in liquid nitrogen using a mortar and pestle.

As previously described [11], total RNA was extracted from forzen kidney powders using RNAiso Plus (9108/9109, Takara, Japan) following the manufacture's protocol. Next, cDNA was synthesized using a Prim-Script™ RT reagent Kit (RR047A, Takara, Japan) according to the manufacture's protocol. The cDNA product was used as a template for qRT-PCR analysis. Sequences for target genes were obtained from the NCBI database. Oligonucleotide primers were designed using Primer 5 software and synthesized at Takara (Dalian, China; Table 2).

**Table 2:** Sequence of primers used in qRT-PCR

Gene symbol	Accession number	Primer	Primer sequence(5′-3′)	Product size	Tm (°C)
ATM	NM001162400.1	Forward Reverse	TTGCCACACTCTTTCCATGT CCCACTGCATATTCCTCCAT	110bp	60
Chk1	AF525027.1	Forward Reverse	GGAAATACCGCCTTGTGTGT CGGAGCTTCTTGTGTTTGAAG	103bp	60
Chk2	NM001080107	Forward Reverse	AGACCAAATCACTCGTGGAGAATAC GATGCTCTAAGGCTTCCTCTATTGT	140bp	60
cdc25	NM001199572.1	Forward Reverse	AGCGAAGATGATGACGGATT GCAGAGATGAAGAGCCAAAGA	163bp	59
p53	NM205264.1	Forward Reverse	ACCTGCACTTACTCCCCGGT TCTTATAGACGGCCACGGCG	127bp	59
p21	AF513031.1	Forward Reverse	TCCCTGCCCTGTACTGTCTAA GCGTGGGCTCTTCCTATACAT	123bp	60
cdc2	NM205314.1	Forward Reverse	TCTGCTCTGTATTCCACTCCTG ATTGTTGGGTGTCCCTAAAGC	144bp	60
cyclinB	NM205239.2	Forward Reverse	ATCACCAACGCTCACAAGAAC AGGCTCCACAGGAACATCTG	171bp	59
PCNA	AB053163.1	Forward Reverse	GATGTTCCTCTCGTTGTGGAG CAGTGCAGTTAAGAGCCTTCC	104bp	60
β-actin	L08165	Forward Reverse	TGCTGTGTTCCCATCTATCG TTGGTGACAATACCGTGTTCA	178bp	62

All qRT-PCR were performed using the SYBR^®^ Premix Ex Taq™II system (DRR820A, Takara, Japan) using on a Model C1000 Thermal Cycler (Bio Rad, USA).

Chicken β-actin expression was used as an internal reference housekeeping gene. Gene expression values from control group subsamples at 14, 28, and 42 days of age were used to calibrate gene expression in subsamples from corresponding experimental subsamples. All data output from the qRT-PCR experiments were analyzed using the 2^−ΔΔCT^ method [64].

### Statistical analysis

The significance of difference among four groups was analyzed by variance analysis, and results presented as mean ± standard deviation ($\bar{X}\pm \text{SD}$). The variation was measured by one-way analysis of variance (ANOVA) test of SPSS 16.0 for windows. Statistical significance was considered at *P* < 0.05.
